# Pharmacokinetics, clearance, and biosafety of polyethylene glycol-coated hollow gold nanospheres

**DOI:** 10.1186/1743-8977-11-26

**Published:** 2014-05-30

**Authors:** Jian You, Jialin Zhou, Min Zhou, Yang Liu, J David Robertson, Dong Liang, Carolyn Van Pelt, Chun Li

**Affiliations:** 1 College of Pharmaceutical Sciences, Zhejiang University, Yuhangtang Road 388, Hangzhou 310058, People’s Republic of China; 2 Department of Cancer Systems Imaging, Unit 59, The University of Texas MD Anderson Cancer Center, Houston, TX 77030, USA; 3 Department of Chemistry, University of Missouri-Columbia, Columbia, MO 65211, USA; 4 Department of Pharmaceutical Sciences, College of Pharmacy and Health Sciences, Texas Southern University, 3100 Cleburne Street, Houston, TX 77004, USA; 5 Department of Veterinary Medicine and Surgery, The University of Texas MD Anderson Cancer Center, Houston 77030, TX, USA

**Keywords:** Hollow gold nanospheres, Toxicity, Photothermal ablation therapy

## Abstract

**Objective:**

Gold nanoparticles have attracted enormous interest as potential theranostic agents. However, little is known about the long-term elimination and systemic toxicity of gold nanoparticles in the literature. Hollow gold nanospheres (HAuNS) is a class of photothermal conducting agent that have shown promises in photoacoustic imaging, photothermal ablation therapy, and drug delivery. It’s very necessary to make clear the biosafety of HAuNS for its further application.

**Methods:**

We investigated the cytotoxicity, complement activation, and platelet aggregation of polyethylene glycol (PEG)-coated HAuNS (PEG-HAuNS, average diameter of 63 nm) *in vitro* and their pharmacokinetics, biodistribution, organ elimination, hematology, clinical chemistry, acute toxicity, and chronic toxicity in mice.

**Results:**

PEG-HAuNS did not induce detectable activation of the complement system and did not induce detectable platelet aggregation. The blood half-life of PEG-HAuNS in mice was 8.19 ± 1.4 hr. The single effective dose of PEG-HAuNS in photothermal ablation therapy was determined to be 12.5 mg/kg. PEG-HAuNS caused no adverse effects after 10 daily intravenous injections over a 2-week period at a dose of 12.5 mg/kg per injection (accumulated dose: 125 mg/kg). Quantitative analysis of the muscle, liver, spleen, and kidney revealed that the levels of Au decreased 45.2%, 28.6%, 41.7%, and 40.8%, respectively, from day 14 to day 90 after the first intravenous injection, indicating that PEG-HAuNS was slowly cleared from these organs in mice.

**Conclusion:**

Our data support the use of PEG-HAuNS as a promising photothermal conducting agent.

## Background

Nanoparticles have great potential for biomedical application, not only to deliver pharmaceutics but also to be used as novel diagnostic and therapeutic agents [1,2]. The difference in the toxicity profiles of bulk materials and their corresponding nanoparticles owing to the tiny physical dimensions of nanoparticles has been widely recognized [3,4]. For example, carbon black is nontoxic; however, carbon nanotubes and fullerene are highly toxic when inhaled into the lung [5,6]. Similarly, the enhanced toxicity of titanium oxide nanoparticles has been reported [7,8], and titanium oxide nanoparticles have been shown to induce oxidative stress in bacteria [9].

Gold nanoparticles (AuNPs) show several features that make them well suited for biomedical applications, including straightforward synthesis, stability, and the ability to selectively incorporate recognition molecules such as peptides or proteins [10]. AuNPs have been used as Raman sensors [11], photocatalysts [12], photoelectrochemical materials [13], photothermal conducting agents [14], biosensors [15], and carriers for the delivery of drugs and genes [16-18]. Although AuNPs are thought to be nontoxic [19-21], there are reports that document their toxicity, which has been shown to depend on the physical dimensions, surface chemistry, and shape of the AuNPs [22-26].

Hollow gold nanospheres (HAuNS) are a novel class of AuNPs composed of a thin Au shell with a hollow interior. Unlike solid AuNPs, HAuNS have plasmon absorption in the near-infrared (NIR) region and display strong photothermal conducting properties suitable for photothermal ablation therapy. HAuNS’ unique combination of small size (30–50 nm in diameter) and a strong, tunable absorption band (520–950 nm) suggests that HAuNS are a promising mediator for a variety of biomedical applications, including imaging and cancer therapy [27-30]. A few studies have explored the potential utility of HAuNS as a novel delivery vehicle to shuttle biomolecules [31,32] or to trigger drug release under NIR light irradiation [33-35]. To date, there has been no detailed study on the toxicity of HAuNS. Herein, we carried out a biosafety evaluation of polyethylene glycol (PEG, molecular weight of 5000)-coated HAuNS (PEG-HAuNS), including *in vitro* blood compatibility and *in vivo* acute and chronic toxicity assessment. We determined the effective therapeutic dose in xenograft models of human ovarian cancer and used the data to guide dose selection for systemic toxicity study.

## Results and discussion

### Synthesis and characterization of HAuNS and PEG-HAuNS

**Figure 1:**
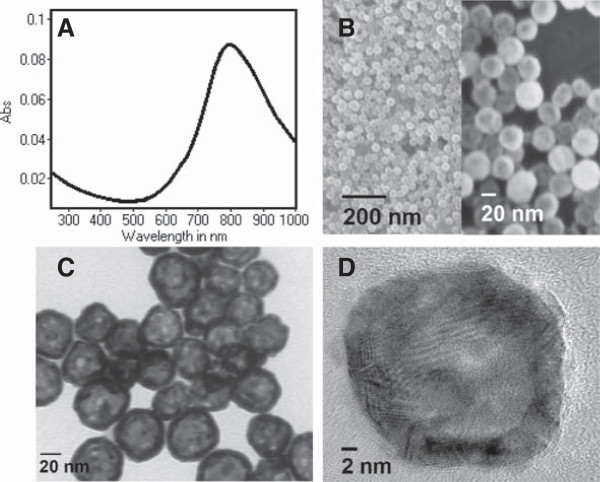
**Characterization of HAuNS. A**: Absorption spectra of HAuNS. **B**: Scanning electron micrograph images of HAuNS. **C** and **D**: Low- and high-resolution TEM images of HAuNS.

HAuNS was readily coated with methoxy-PEG-sulfhydryl (molecular weight, 5,000). The absorption spectra showed that the plasma resonance peak for PEG-HAuNS was tuned to the NIR region (~800 nm) (Figure 1A). The homogeneity of HAuNS is demonstrated in Figures 1B and C, which show representative scanning electron micrograph and low-resolution TEM images of HAuNS. The narrow size distribution of the HAuNS resulted from the narrow size distribution of the cobalt seed nanoparticles from which the HAuNS were grown. The high-resolution TEM of an individual HAuNS, shown in Figure 1D, illustrates the thin Au shell with hollow interior. TEM reviewed an average size of 43.1 ± 5.4 nm (Figure 1). Dynamic light scattering revealed an average HAuNS diameter of 43.6 nm, and average polydispersity of 0.006. PEGylation led to a significant increase in the size of PEG-HAuNS, which had an average diameter of 62.7 nm and polydispersity of 0.068. However, compared with HAuNS, PEG-HAuNS had significantly increased colloidal stability: no aggregation was observed when PEG-HAuNS were stored in water at room temperature over a period of 6 months. PEG modification did not affect the extinction spectrum of HAuNS. The physicochemical properties of HAuNS and PEG-HAuNS are summarized in Table 1.

**Table 1:** Physicochemical properties of HAuNS and PEG-HAuNS

	**HAuNS**	**PEG-HAuNS**
Concentration (mg/mL)	2.5 × 10^−3^	2.5 × 10^−3^
Concentration (particles/mL)	6.25 × 10^9^	6.25 × 10^9^
Mean diameter^a^ (nm)	43.6 ± 0.4	62.7 ± 0.7
Polydispersity^a^	0.006 ± 0.001	0.068 ± 0.005
Zeta potential (mA)	−23 ± 0.8	−15 ± 0.6
Plasma resonance peak (nm)	802	801
Optical density^b^	0.112	0.110

^a^Data are presented as mean ± standard deviation (n = 3), measured by dynamic light scattering. ^b^Optical density measured at 800 nm.

### Cytotoxicity

The cytotoxicity of PEG-HAuNS against both LLC-PK1 and HepG2 cells increased with increased incubation time (Additional file 1: Figure S1). PEG-HAuNS was more toxic to kidney cells than to liver cells (HepG2): the IC_50_ values of PEG-HAuNS after 48 h of incubation in LLC-PK1 and HepG2 cells were 0.22 mg/mL and >1 mg/mL, respectively.

On the basis of cytotoxicity assay, gold nanoparticles have been found to be “non-toxic” or “toxic”, depending on the physicochemical characteristics (surface charge, size, and surface chemistry) of nanoparticles [26]. A direct comparison of our results with those reported in the literature would not be possible because of differences in nanoparticle characteristics and cell lines used in these studies. Nevertheless, Patra el al. [36] found that gold nanoparticles of 33 nm in diameter, which was only slightly smaller than our HAuNS, were not toxic to Hep2G and BHK21 (baby hamster kidney) cells. In our cytotoxicity study, concentrations of 0.22 mg/mL and > 1 mg/mL for LLC-PK1 and Hep2G cells respectively were quite high. At such concentrations, absorption at 495 nm by residual nanoparticles may affect MTT assay results as suggested by Alkilany et al. [26]. Because we have observed that the decrease in optical density as a function of particle dose depended on incubation time, suggesting that the effect of residual nanoparticles on the MTT assay was not significant.

### Complement activation and platelet aggregation

**Figure 2:**
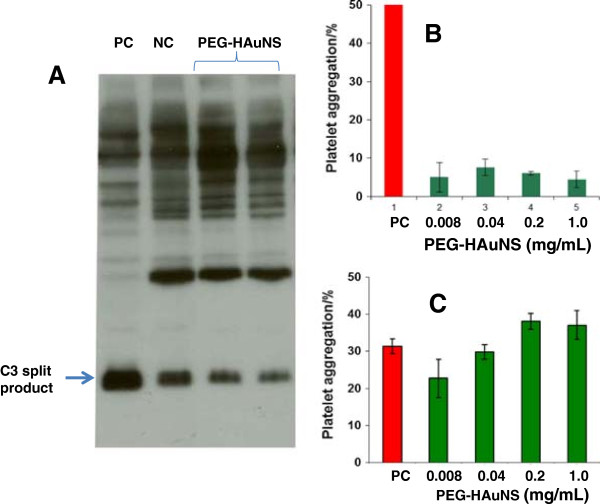
***In vitro *complement activation and platelet aggregation assays. A**: Complement activation assay of PEG-HAuNS (1 mg/mL). The nanoparticles did not result in the detectable activation of a complement system. PC, positive control (cobra venom), NC, negative control (PBS). **B**: Platelet aggregation in the presence of PEG-HAuNS. PEG-HAUNS under tested concentrations did not induce platelet aggregation. **C**: PEG-HAUNS under tested concentrations did not interfere with collagen-induced platelet aggregation. PC, positive control (collagen).

To examine potential effects of PEG-HAuNS on the complement activation cascade, we assessed the degree of complement activation in an *in vitro* assay in human plasma aliquots pretreated with nanoparticles. PEG-HAuNS did not induce detectable activation of the complement system in a C3-specific qualitative assay (Figure 2A). Furthermore, PEG-HAuNS under the tested concentrations did not induce any detectable platelet aggregation (Figure 2B) and did not interfere with collagen-induced platelet aggregation (Figure 2C).

### Pharmacokinetics and biodistribution

**Figure 3:**
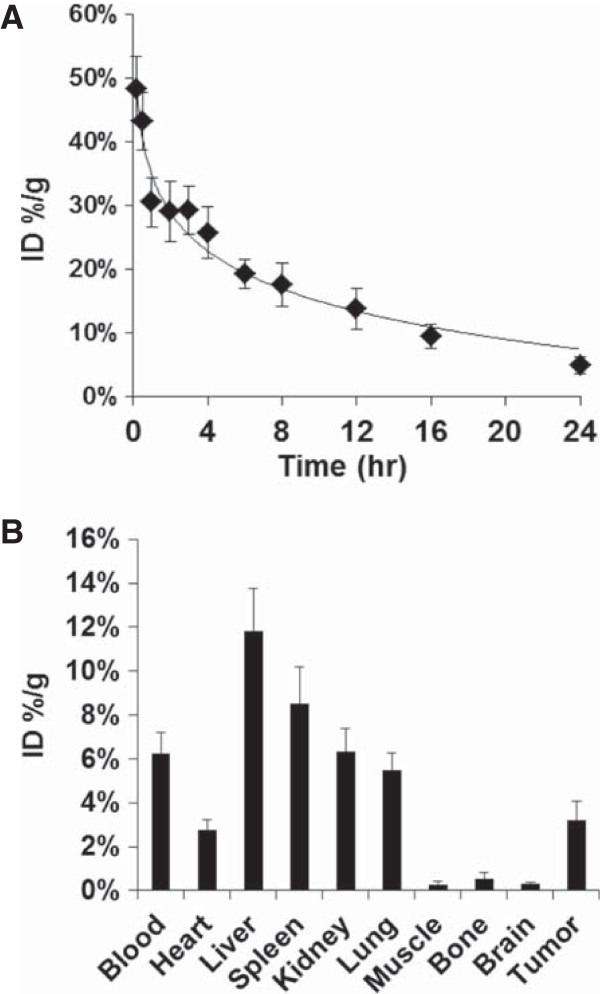
**Pharmacokinetics and biodistribution of PEG-HAuNS. A**: Blood level profiles of ^64^Cu-labeled PEG-HAuNS. The data are expressed as a percentage of the injected dose (5.0 mL/kg of 50 OD PEG-HAuNS, 1.25 mg/mL) per gram of blood (%ID/g) and are presented as mean ± standard deviation (n = 8). **B**: Biodistribution of ^64^Cu-labeled PEG-HAuNS in mice at 24 h after intravenous injection (n = 6).

Figure 3A shows the mean blood level profile of ^64^Cu-labeled PEG-HAuNS. Terminal biological half-life, apparent volume of distribution, total body clearance, mean residence time, and total area under the blood concentration were 8.19 ± 1.4 hr, 2.78 ± 0.63 mL, 0.235 ± 0.03 mL/hr, 12.3 ± 2.2 hr, and 434 ± 61%ID · hr/mL, respectively. Figure 3B shows the biodistribution of PEG-HAuNS in Hey tumor-bearing mice at 24 h after intravenous injection. Most nanoparticles were taken up by the liver (11.8 ± 1.95%ID/g) and spleen (8.51 ± 1.67%ID/g). Because blood was not perfused prior to measurements in our study, the uptake values in the liver and spleen may be overestimated owing to the contribution of PEG-HAuNS in the blood in these organs. A significant amount of PEG-HAuNS also accumulated in the tumors (3.22 ± 0.86%ID/g), which could be attributed to the enhanced permeability and retention effect.

Tissue distribution of PEGylated gold nanoparticles have been reported by several groups. A direct comparison of our results with those reported in the literature is difficult because particle characteristics (size, surface chemistry, charge etc.) of these gold nanoparticles are very different. Lankveld et al. [37] found that spleen was the major organ of particle deposition in rats for PEGylated gold nanorods (55.3 × 18.5 nm). Similar finding was reported by Akiyama et al. [38] using PEGylated nanorods of 55 × 9 nm in average size in mice. Interestingly, a PEGylated gold nanorod preparation with slightly different size (65 × 11 nm) showed mostly liver uptake and minimal spleen uptake 24 h after intravenous injection in mice [39]. We have found a size-depend distribution pattern for spherical gold nanoparticles coated with PEG. Thus, PEGylated gold nanoparticles of smaller size (20-nm in diameter) had longer blood half-life and lower uptake in the liver and spleen than those of larger size (80-nm in diameter) [40]. In agreement with the current findings, PEGylated gold nanoparticles were largely distributed to the liver and the spleen [40].

### Antitumor activity

**Figure 4:**
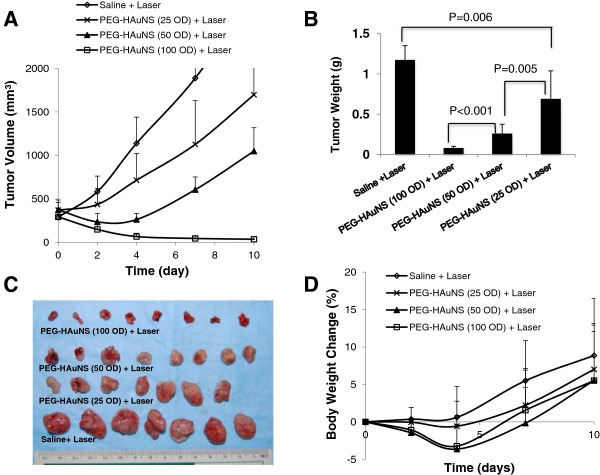
**Antitumor activity of PEG-HAuNS against Hey tumors in nude mice. A**: Hey tumor growth curves in mice treated with saline (n = 7), and PEG-HAuNS at doses ranging from 5 mL/kg of 25 OD (n = 7, 3.125 mg/kg), 50 OD (n = 8, 6.25 mg/kg), and 100 OD (n = 8, 12.5 mg/kg). All tumors received NIR laser illumination from the tumor’s surface (2.5 W/cm^2^, for 3 min) at 24 h after a single dose nanoparticle injection. **B**: Average weight of tumors in saline- and PEG-HAuNS-treated groups on day 10 after NIR laser illumination. **C**: Photographs of excised tumors on day 10 after NIR laser illumination. **D**: Percentage change in mean body weight.

To facilitate selection of a more realistic dosing schedule for assessment of toxicity of PEG-HAuNS, we first determined the effective dose of PEG-HAuNS in NIR laser-induced photothermal ablation therapy in a human ovarian tumor model (Figure 4). Results from Hey tumor-bearing nude mice showed a dose–response to PEG-HAuNS at a laser output power of 2.5 W/cm^2^ for 3 min of continuous illumination. At a single dose of 5.0 mL/kg of 100 OD (12.5 mg/kg), PEG-HAuNS displayed the greatest antitumor activity (Figure 4A and B). All of the tumors in this group shrank to small lumps, which were barely palpable (Figure 4C). Average body weight of the mice in the 50-OD and 100-OD dosing groups decreased after NIR laser illumination, with about 5% body weight loss on day 4 after the illumination. No change in body weight for the mice in both the saline and 25-OD dosing groups was observed during the same period (Figure 4D). The body weight of all treated groups started to recover 4 days after laser illumination.

Additional file 1: Figure S2 shows the A2780 tumor-growth curves after intravenous injection of saline or PEG-HAuNS (single dose, 5.0 mL/kg of 50 OD, 6.25 mg/kg), followed by NIR laser treatment (2.5 W/cm^2^ for 3 min) at 24 h after injection. PEG-HAuNS followed by NIR laser treatment significantly inhibited the growth of the tumors. The average tumor weight in this group on day 21 was 0.1 ± 0.02 g, which was significantly smaller than that of the saline and laser-treated control groups (2.0 ± 0.4 g) (*p* < 0.001). Thus, a single dose of PEG-HAuNS ranging from 6.25 mg/kg to 12.5 mg/kg were effective in mediating photothermal ablation therapy.

### Acute and chronic toxicity

The available literature on *in vitro* and *in vivo* toxicity of AuNPs showed contradicting findings, primarily because *in vivo* behaviors of AuNPs are dependent on their physicochemical characteristics [26]. Little is known about AuNPs’ elimination from major organs and their long-term biosafety after intravenous injection [26]. In this context, there is a real need to investigate the *in vivo* toxicity of gold-based nanoparticles intended for therapeutic use, in particular after long-term exposure (>60 days). On the basis of the therapeutic studies, systemic toxicity study of PEG-HAuNS was conducted at an intravenous dose of 12.5 mg/kg per injection, for a total of 10 injections administered over a 2 week period. No clinical signs were observed during the course of the study, and all animals survived until their scheduled termination. No toxicologically important alterations in body weights and relative organ weights were observed at either time point (day 14 and day 90 after start of treatment) (See Additional file 1: Tables S1 and S2 for specific group means and calculated percent of control).

#### Hematology

A complete blood count and clinical chemistry parameters were performed on the cardiac blood collected at the time of necropsy for both 14-day and 90-day time points. Table 2 and Additional file 1: Tables S3 summarize the group means for each group and the laboratory reference range for 6- to 8-week-old CD-1 mice using the same instruments used in this study. Differences in the mean values of the PEG-HAuNS-treated groups are highlighted if there were alterations from their respective concurrent control group.

**Table 2:** Group means for both sacrifice dates and both sexes for clinical chemistry

	Parameter*	**Creatinine**	**BUN**	**AST**	**ALT**	**ALK PHOS**	**T. Protein**	**Albumin**	**Globulin**
	**Unit**	**mg/dL**	**mg/dL**	**mg/dL**	**IU/L**	**IU/L**	**IU/L**	**g/dL**	**g/dL**
14 days (Female)	Saline	<0.20	16.1	443	259	128	4.91	3.63	1.29
	PEG-HAuNS	<0.20	14.3	515	316	136	5.18	3.59	1.58
90 days (Female)	Saline	<0.20	16.9	273	130	92	5.8	3.61	2.19
	PEG-HAuNS	<0.20	16.4	406	202	84	5.88	3.75	2.13
14 days (Male)	Saline	<0.20	18	112	69	123	5.63	3.55	2.07
	PEG-HAuNS	0.2	19.8	322	256	123	5.52	3.33	2.19
90 days (Male)	Saline	0.22	20.3	152	109	137	6.62	3.83	2.79
	PEG-HAuNS	0.27	17.6	591	395	204	6.62	3.76	2.85
Reference ranges	Males	0-0.4	23-38	0-541	0-561	110-219	5.1-6.2	2.5-3.2	2.5-3.0
	Females	0-0.4	19-33	0-671	0-596	113-223	5.3-6.3	2.8-3.5	2.4-2.9

*BUN - blood urea nitrogen, *AST* - aspartate aminotransferase, *ALT* - alanine aminotransferase, *ALK PHOS* - alkaline phosphatase, *T*. protein - total protein.

The only alterations in hematology (using concurrent controls) occurred in females at 14 days. The total white blood cell, absolute lymphocyte, and eosinophil counts were slightly elevated but returned to normal by the 90-day time point. These alterations were minimal and not considered toxicologically important.

#### Clinical chemistry

The chemistry parameters evaluated were creatinine, blood urea nitrogen, aspartate aminotransferase (AST), alanine aminotransferase (ALT), alkaline phosphatase, total protein, albumin, and globulin.

The AST and ALT group means were increased in both sexes at both day 14 and day 90, compared to their respective controls. However, none of the groups, except the males at the 90-day treatment time point, had an increase above the reference range of the laboratory. Examination of the individual animal data for these 2 parameters indicated that mice in all the groups had samples with severely hemolyzed sera that can falsely elevate both AST and ALT. When the values for AST and ALT from animals with severe hemolysis were eliminated from computation of the group means, no increase above the reference range for this laboratory was observed. On the basis of this analysis, this alteration in these 2 parameters appeared spurious, and related to sample quality, i.e., hemolysis. This interpretation was supported by normal values for the other clinical chemistry parameters measured to evaluate the liver, i.e., total protein, albumin, and globulin levels. This interpretation was also supported by the relative organ weight data for the liver. Correlation with the histopathology data showed very minimal focal necrosis and minimal deposit of pigment in occasional hepatocytes that could potentially increase both ALT and AST.

#### Gross pathology

PEG-HAuNS-related gross pathological findings observed in the PEG-HAuNS-treated group on day 14 were limited to liver lesions. This lesion was recorded as a discoloration of the liver in all test animals from both sexes. This discoloration correlates with pigment observed in the Kupffer cells of the liver on microscopic examination. No gross lesions were observed in any of the animals at day 90.

#### Microscopic pathology

Summary incidences and average group grades for all microscopic observations at both day 14 and day 90 were presented in Additional file 1: Tables S4. The major microscopic observation in the PEG-HAuNS-treated groups was deposition of a brown-black pigment in the liver, spleen, lungs, heart, adrenal cortex, and injection site. The largest amounts of pigment were observed in the liver and spleen. All treated mice had deposition of pigment in the liver (24/24) and 16/24 treated mice had pigment deposited in the spleen. Pigment was more prevalent at 14 days and had diminished by 90 days in these 2 organs. The incidence of this pigment in the other organs was as follows: lung (2/24), heart (7/24), adrenal cortex (3/24), and injection site at the tail vein (3/24). It was minimal in quantity, frequently occurred in perivascular areas in these organs, and generally was not associated with inflammatory or degenerative changes. It is not clear why PEG-HAuNS stayed at perivascular areas. One possible explanation is that these nanoparticles came from blood during tissue processing because blood was not perfused at the time of euthanization and tissue harvesting. Another possibility is that some of the nanoparticles were able to penetrate endothelial lining and trapped in the perivascular area. Further studies are needed to clarify this interesting observation.

The major target organs were considered the liver and spleen, and the monocytic-macrophage system was the primary target.

##### Liver

**Figure 5:**
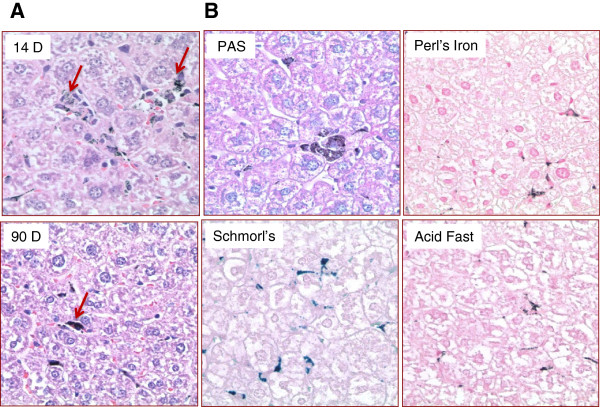
**Liver pigment. A**: Arrows point to pigment in both 14-day and 90-day liver tissues. The 14-day tissue has more Kupffer cells filled with pigment. The 90-day tissue has pigment but fewer Kupffer cells with pigment. **B**: Section of liver pigment stained with PAS, Perl’s iron, Schmorl’s, and acid fast. Schmorl's stain has some of the slightly blue reaction for lipofuscin, but the other lipofuscin stains (PAS, acid fast) do not have the characteristic color for lipofuscin. The Perl’s iron stain is negative for iron. Magnification: ×400.

**Figure 6:**
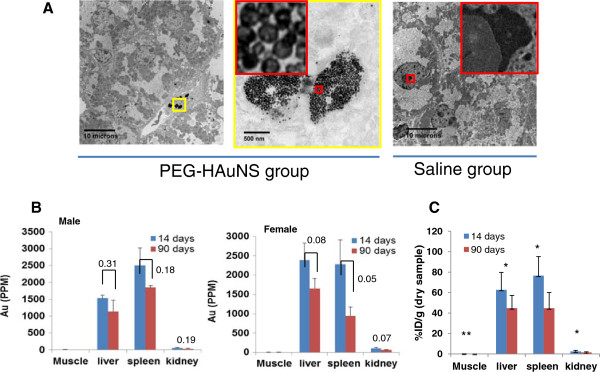
**Clearance of Au nanoparticles from mice. A**: Representative transmission electron microscopy images of liver tissues from PEG-HAuNS- and saline-treated mice at the 14-day time point. Au nanoparticles and pigment were found only in the PEG-HAuNS-treated mice, indicating that the pigment in the macrophages of the liver (Kupffer cells) was attributed to PEG-HAuNS. **B**: Au concentration (ppm) in various dried tissue samples from male (n = 3) and female (n = 3) mice at day 14 and day 90 after the first injection of PEG-HAuNS. The p values (two tailed student’s *t*-test) are provided in the graphs. **C**: Tissue concentration of Au expressed as percentage of injected dose per gram drug tissue (%ID/g) obtained from both female and male mice (n = 6). ** p < 0.01; * p < 0.05.

In both female and male mice, a dark, brown-black pigment was observed, primarily in the macrophages (Kupffer cells) of the liver and occasionally in a few hepatocytes. This pigment was present in nearly all Kupffer cells at the 14-day time point in females and males, but there was a decrease in the number of Kupffer cells affected and a slight decrease in the quantity (lesion grades) of the pigment at the 90-day time point in both females and males (Figure 5A). Special stains (Perl’s iron, Schmorl’s stain for lipofuscin, Periodic Acid-Schiff [PAS], and acid-fast) (Figure 5B) were done to characterize this pigment. The Schmorl’s stain was positive in the liver and spleen, but the other stains to confirm lipofuscin (PAS, acid-fast) were negative, indicating the pigment was not lipofuscin. Samples of formalin-fixed liver were submitted for transmission electron microscopic study (TEM) and confirmed that the pigment observed in the Kupffer cells and some hepatocytes was electron-dense nanospheres comparable to the injected PEG-HAuNS (Figure 6A).

To further confirm liver uptake of PEG-HAuNS, samples of liver were submitted for Au quantification using neutron activation analysis (control and treated at both time points). Au was only detected in the treated samples. Although there was a general trend of decreasing concentration from day-14 to day-90 for muscle, liver, spleen, and kidney in both male (n = 3) and female mice (n = 3) (Figure 6B), the differences were not statistically significant with the exception of spleen in female mice (p = 0.05). However, when data from both male and female mice are combined (n = 6), significant differences in tissue concentration of Au for all organs analyzed (muscle, liver, spleen, and kidney, with p values of 0.006, 0.027, 0.011, and 0.015, respectively) were demonstrated (Figure 6C). Thus, the levels of Au in the muscle, liver, spleen, and kidney decreased 45.2%, 28.6%, 41.7%, and 40.8%, respectively, from day 14 to day 90 after the first intravenous injection (Table 3). These data suggest that PEG-HAuNS could be cleared from the body, albeit at a very slow rate.

**Table 3:** Percentage decrease in gold concentration in various organs for male and female mice from day 14 to day 90

	**Muscle**	**Liver**	**Spleen**	**Kidney**
Males (n = 3)	44.6	25.2	26.2	38.8
Females (n = 3)	45.7	30.8	58.6	41.8
Males & females (n = 6)	45.2	28.6	41.7	40.8

Both inflammation and necrosis were minimal in the livers. Inflammation occurred in both control and treated animals of both sexes at both time points, but there was a slightly higher incidence and minimally increased severity in the treated animals. At day 90, the incidence and severity decreased slightly in females but increased slightly in males. The severity grade was slight and not considered adverse. Focal necrosis was observed in female mice only at the 90-day time point, with an incidence of 2 (out of 6 mice) and an average grade of 1. In male mice, focal necrosis was observed at the 14-day time point, with an incidence of 3 of 6 and a grade of 1, but not at the 90-day time point. Since an incidence of only 1 was observed in the male controls at day 14, there was a very slight increase in focal necrosis in both females and males; however, the time of occurrence did not correlate between the sexes. Thus, this is a minimal increase over controls and not considered adverse.In summary, the major hepatic lesion was a deposition of pigment in the macrophages of the liver (Kupffer cells). The pigment was identified as a deposition of the Au nanospheres by both TEM and quantitative analysis of selected formalin-fixed samples of liver from this study (Figure 6). Comparison of pigment in histopathology samples between groups and quantitative analysis for Au indicate that the deposited pigment was greatest at the 14-day time point and was decreasing at the 90-day time point, indicating slow clearance at 90 days. Minimal hepatic lesions of inflammation and necrosis were observed, but the incidence and severity were minimal in both sexes and not considered adverse. The increase and continued presence of mononuclear inflammation in both sexes suggest these cells may be recruited macrophages for the removal of pigment, a normal biologic response that does not have adverse effects.

##### Spleen

A dark brown-black pigment consistent with the pigment observed in the liver was observed in the macrophages of the spleen in the treated animals at both time points. At 14 days the pigment deposition was small and diffusely throughout the spleen. At 90 days, the pigment was larger but fewer aggregates distributed focally and randomly throughout the spleen (Additional file 1: Figure S3). Based on the histopathologic analysis and the Au quantification (Figure 6B), this pigment was interpreted to be the injected PEG-HAuNS. Its presence in the macrophages of the spleen is considered a normal biologic response of removing foreign material and not adverse. An increase in follicular lymphoid hyperplasia and extramedullary hematopoiesis was observed in PEG-HAuNS-treated animals compared to the respective controls, but the incidence and severity was minimal, considered a normal biologic response to injection of foreign material, and not adverse.

##### Lung

**Figure 7:**
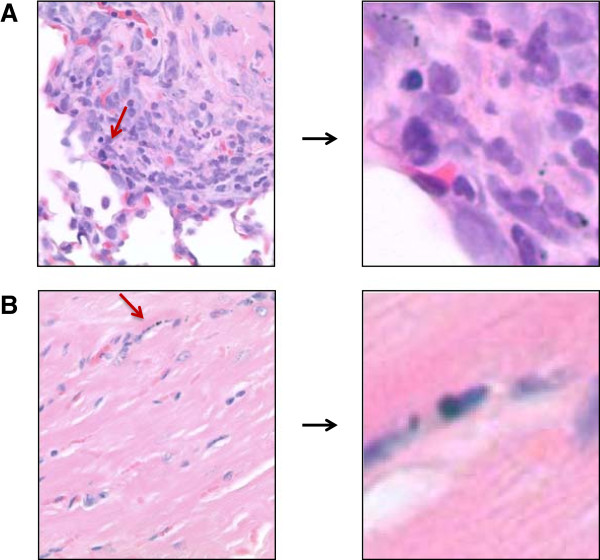
**Pigment in lung and heart. A**: The image on the left is from a 14-day-PEG-HAuNS-treated animal with peribronchiolar inflammation. Arrow points to pigment. **B**: Arrow points to pigment in lung tissue from a 90-day-PEG-HAuNS-treated animal. Magnification: ×400.

Several types of inflammation were observed in the lungs; these inflammatory lesions included mononuclear (histiocytic) cells and granuloma formation. These lesions were more prevalent in the treated animals than in the controls. In the treated animals, a dark brown-black pigment was found in a few of the inflammatory lesions (Figure 7A). In the female mice, the incidence of the lesions was higher at the 14-day time point when considering all lesions and decreased at the 90-day time point, indicating recovery. In the male mice, the lesions were fewer and less severe but persisted to the 90-day time point. Special stains (PAS, Perl’s iron, Schmorl’s stain for lipofuscin, acid fast) did not identify any foreign material in the granulomas. All lesions except 1 were grade 1, indicating minimal involvement. On the basis of this minimal involvement, the lesions were not considered adverse. While some of these lesions may or may not be related to treatment, they are minimal severity, and low incidence and not considered to have any significant impact on the health of the animals with them.

##### Heart

Murine cardiomyopathy occurs in mice and the morphologic changes include myocyte degeneration/necrosis, an increase in the amount of interstitial fibrous connective tissue, and in some cases a minimal to mild infiltrate of mononuclear inflammatory cells. These age-related cardiomyopathy have been reported in different strains of laboratory mice [41]. Inflammatory and degenerative lesions typical of spontaneous cardiomyopathy (mononuclear inflammation, myocyte degeneration, fibrosis) were observed in both the treated and control animals. However in the treated mice, these lesions were sometimes associated with very small deposits of dark brown pigment similar to that observed in other organs. This pigment was observed in 7/24 treated animals (Figure 7B) but in none of the 3/24 controls that had cardiac lesions. These lesions would have been classified as spontaneous cardiomyopathy if the pigment had not been present. Since pigment is not typical of cardiomyopathy, these lesions were tabulated individually and not included under the cardiomyopathy that was used in the controls. Special stains did not identify the pigment, and all heart tissue was used in the histopathologic analysis. The pigment deposit in each case was minimal and generally a single observation in the sections of the heart examined. What cannot be explained is whether the pigment deposition was incidental to the lesions or whether its presence contributed to the development of the lesions. On the basis of the number and size of the lesions, these lesions were not considered adverse. They may or may not be related to the compound, but are not of the severity or frequency to impact the health of the animal.

##### Adrenal

The adrenal gland in only the female mice had an increase in vacuolation of the cortex and pigment deposition in the cortex at the 14-day time point in the treated animals but not in their respective controls. By 90 days, the treated females had less vacuolation in the cortex than their respective controls, but the pigment deposition remained. Vacuolation of the adrenal cortex in the female mice occur as a result of the spontaneous regression of the X-zone of the cortex and its replacement with fat that can undergo lipogenesis (formation of lipid pigments such as lipofuscin). Special stains for lipofuscin were negative on the pigment in the adrenal cortex. Since the morphology of the pigment was similar to that observed in the heart and liver, it was presumed to be HAuNS. As observed in the heart, the pigment was minimal at best and usually only a single observation in the tissue section. Its association with a normal spontaneous process (regression of the X-zone) may be incidental. On the basis of the size of the deposits, this lesion was not considered adverse.

##### Injection site

Deposition of a dark brown pigment similar to that observed in other tissues and identified as HAuNS occurred in the perivascular area and the wall of vessels at the injection site at the tail vein in 3/24 treated animals — 2 in the 14-day group and 1 at 90 days. On the basis of the size of the lesions and frequency of tissue reaction to the pigment, it was not considered adverse.

##### Other organs

Microscopic lesions were observed in other organs: kidney, brain, eyes (retina), spinal cord, sciatic nerve, intestinal tract, salivary glands, and associated lymph nodes and thyroid. These observations occurred in both the control and treated animals and did not have an increased incidence in treated animals compared to controls. These lesions are common observations in this strain of laboratory mouse and are considered incidental and spontaneous in this study.

## Conclusion

In this work, we investigated the cytotoxicity, blood compatibility, pharmacokinetics, biodistribution, and systemic toxicity of PEG-HAuNS. *In vitro*, PEG-HAuNS did not induce detectable complement activation and platelet aggregation. Under the conditions of this study and on the basis of clinical signs, survivability, clinical pathology, and total body and relative organ weights and pathology, the administration of PEG-HAuNS was not considered adverse in female or male mice at an accumulated injection dose of 125 mg/kg, which was 10-fold higher than the effective therapeutic dose. Pathologic and quantitative analysis for Au in formalin-fixed tissues indicated that the levels of deposited HAuNS were decreased at 90 days, but HAuNS was not completely eliminated. The primary test-substance-related microscopic observation in this study was the deposition of pigment, primarily in the macrophages of the liver and spleen that was identified as HAuNS based on TEM and Au quantification. It is interesting to note that the increase and continued presence of mononuclear inflammation in the liver suggest these cells may be recruited macrophages for the removal of pigment, a normal biologic response that does not have adverse effects. Minute deposits of pigment (assumed to be HAuNS) were observed in the lung, heart, and adrenal cortex (only in females) and at the injection site. Importantly, quantitative analysis for Au in the liver, spleen, and kidney revealed that the levels of deposited PEG-HAuNS gradually decreased from day 14 to day 90 after the first injection, indicating that PEG-HAuNS was gradually cleared from the body, albeit slowly. Completely eliminating Au from the body may take a much longer time. Taken together, our data support the use of PEG-HAuNS as a potential photothermal conducting agent.

## Methods

### Reagents

Methoxy-PEG-sulfhydryl (molecular weight, 5,000) was purchased from Nektar (San Francisco, CA). (3-(4,5-Dimethylthiazol-2-yl)-2,5-diphenyltetrazolium bromide (MTT) and 4,6-diamidino-2-phenylindole (DAPI) were purchased from Sigma-Aldrich (St. Louis, MO). Trisodium citrate dehydrate (>99%), cobalt chloride hexahydrate (99.99%), sodium borohydride (99%), and chloroauric acid trihydrate (American Chemical Society reagent grade) were purchased from Fisher Scientific (Pittsburgh, PA) and were used as received. ^64^CuCl_2_ radionuclide was obtained from Washington University (St. Louis, MO).

### Cell culture

Hey (human ovarian carcinoma) cells were purchased from the American Type Culture Collection (Manassas, VA). The Hey cells were maintained at 37°C in a humidified atmosphere containing 5% CO_2_ in Dulbecco’s modified Eagle’s medium (DEME) and 10% fetal bovine serum (FBS, Life Technologies, Inc., Grand Island, NY). A2780 human ovarian carcinoma cells were kindly provided by Dr. Stephen J. Williams (Fox Chase Cancer Center, Philadelphia, PA) and maintained at 37°C in RPMI-1640 medium containing 10% FBS and insulin (0.25 units/mL). LLC-PK1, an epithelial porcine kidney cell line with proximal tubule properties, was obtained from the American Type Culture Collection and was grown in DEME (1.0 g/L glucose) supplemented with 10% FBS, 1% glutamine, 25 mmol/L (4-(2-hydroxyethyl)-1-piperazineethanesulfonic acid) buffer, and antibiotics. Cells were divided twice a week to ensure exponential growth and were cultured for no more than 10 passages after thawing them from stock. The human hepatocellular liver carcinoma cell line HepG2 (American Type Culture Collection) was cultured in DENE with 10% FBS and antibiotics.

### Synthesis and characterization of HAuNS and PEG-HAuNS

HAuNS were synthesized according to a previously reported method [34]. Briefly, cobalt nanoparticles were first synthesized by deoxygenating deionized water containing 4.5 mL of 1-mol/L sodium borohydride, 2.8 mL of 0.1-mol/L sodium citrate, and 1.0 mL of 0.4-mol/L cobalt chloride. After chloroauric acid was added to the solution containing the cobalt nanoparticles, the cobalt immediately reduced the Au ions onto the surface of the cobalt nanoparticles, while at the same time it was oxidized to cobalt oxide. Any remaining cobalt core was further oxidized by air, resulting in the final product, HAuNS. The size of the HAuNS was determined using dynamic light scattering on a Brookhaven 90 plus particle size analyzer (Holtsville, NY). Ultraviolet–visible spectroscopy was recorded on a Beckman Coulter DU-800 ultraviolet–visible spectrometer (Fullerton, CA). The morphology of the HAuNS was examined using a JEM 1010 transmission electron microscope (JEOL USA, Inc., Peabody, MA). For the preparation of PEG-HAuNS, briefly, 2 mL of HAuNS (100 optical density [OD]) were added to argon-purged aqueous solution containing PEG sulfhydryl (3 μmol). The reaction was allowed to proceed overnight at room temperature. For purification, the reaction mixture was centrifuged at 10,000 rpm for 15 min, and the resulting pellet was resuspended in deionized water. The process was repeated twice to remove any unattached PEG molecules.

### ^64^Cu radiolabeling of PEG-HAuNS

For conjugation of the radiometal chelator to PEG-HAuNS, 1,4,7,10-tetraazacyclododecane -*N, N', N'', N'''*-tetraacetic acid thioctamide (DOTA-TA, 1.0 mg/mL; 20 μL) was mixed with 1.0 mL of aqueous solution of HAuNS (160 OD/mL) for 6 h at room temperature. Unreacted DOTA-TA was removed by centrifugation at 10,000 rpm for 10 min. Then DOTA-conjugated HAuNS was further reacted with PEG sulfhydryl to obtain DOTA-conjugated PEG-HAuNS. Conjugating PEG to HAuNS first followed by introduction of DOTA was proven to be less efficient perhaps because the steric barrier formed by PEG chains could prevent DOTA-TA from approaching the surface of HAuNS. For radiolabeling with ^64^Cu, aliquots of DOTA-conjugated PEG-HAuNS (160 OD/mL) in 0.1-M sodium acetate solution (pH 5.4) were mixed with an aqueous solution of ^64^CuCl_2_ (10 mCi) for 1 h. The radiolabeled nanoparticles were then purified by centrifugation at 10,000 rpm for 10 min and washed 3 times with phosphate-buffered saline. The nanoparticles were resuspended in phosphate-buffered saline. The radiolabeling efficiency and the stability of labeled nanoparticles were analyzed using instant thin-layer chromatography. The paper strips were developed with phosphate-buffered saline (pH 7.4) containing 4.0 mM ethylenediaminetetraacetic acid, and the radioactivity was quantified using a Bioscan IAR-2000 thin-layer chromatography imaging scanner (Washington, DC). Free ^64^Cu^2+^ moved to the solvent front (Rf = 0.9-1.0), and the nanoparticles remained at the original spot (Rf = 0.0). The labeling efficiency was > 95%.

### Transmission electron microscopy (TEM)

The tissue uptake of PEG-HAuNS was studied by TEM. Briefly, sections of liver tissue from a control mouse and a male mouse at 14-day time point were fixed with a solution containing 3% glutaraldehyde plus 2% paraformaldehyde in 1.0 M cacodylate buffer (pH 7.3) for 1 h. After fixation, the samples were washed and treated with 0.1% Millipore-filtered (Billerica, MA), cacodylate-buffered tannic acid, post-fixed with 1% buffered osmium tetroxide for 30 min, and stained en bloc with 1% Millipore-filtered uranyl acetate. The samples were dehydrated in increasing concentrations of ethanol, infiltrated, and embedded in Ladd-112 medium. The samples were polymerized in an oven at 70°C for 2 days. Ultrathin sections were cut in a Leica Ultracut microtome (Deerfield, IL), stained with uranyl acetate and lead citrate in a Leica EM stainer, and examined in a JEM 1010 transmission electron microscope (JEOL USA, Inc.) at an accelerating voltage of 80 KV. Digital images were obtained using the AMT Imaging System (Advanced Microscopy Techniques Corp, Danvers, MA).

### Cytotoxicity

Cytotoxicity was measured using the MTT assay according to the manufacturer’s suggested procedures. LLC-PK1 and HepG2 cells were exposed to PEG-HAuNS at various concentrations for 4, 24, and 48 h. The data are expressed as percentage of survival cells and are reported as the means of triplicate measurements in a single experiment.

### Complement activation

Platelet-poor plasma was prepared by centrifugation of freshly drawn whole human blood for 10 min at 2,500 *g* and was used fresh. Complement activation experiments were performed as described previously (http://ncl.cancer.gov/NCL_Method_ITA-5.pdf). Briefly, equal volumes (10 μL) of test samples (PEG-HAuNS at final concentration of 1 mg/mL, cobra venom factor at concentration of 50 U as positive control, or PBS as negative control), freshly prepared plasma, and veronal-buffer were mixed together and incubated at 37°C for 60 min. The reaction was stopped by the addition of 4 × NuPAGE sample buffer (Invitrogen, Carlsbad, CA). After heating for 5 min at 90°C, 30 μL of the sample were resolved on 10% Trisglycine gel. For Western blot analysis, proteins separated by 2-dimensional polyacrylamide gel electrophoresis were transferred onto a Westran S nylon (Whatman Inc., Florham Park, NJ) membrane and probed with goat polyclonal antibodies specific to C3 component of complement (Calbiochem, San Diego, CA).

### Platelet aggregation

To study the nanoparticles’ effects on platelet aggregation, whole human blood was centrifuged 8 min at 200 *g* to obtain platelet-rich plasma, which was then treated with PEG-HAuNS (0.008, 0.04, 0.2, and 1.0 mg/mL) or collagen (positive control; Helena Laboratories, Beaumont, TX) for 15 min at 37°C. To investigate whether PEG-HAuNS can interfere with collagen-induced platelet aggregation, platelet-rich plasma was also treated with the mixture of collagen and PEG-HAuNS under the same conditions. A single-platelet count was then conducted using a Z2 counter and size analyzer (Beckman Coulter, Inc.). A decrease in the single-platelet count occurring due to the platelet aggregation was used to calculate percentage aggregation. A detailed protocol is available at http://ncl.cancer.gov/NCL Method ITA-2.pdf.

### Pharmacokinetics and biodistribution

All animal studies were carried out at MD Anderson Cancer Center (MDACC) under Institutional Animal Care and Use Committee-approved protocols. All animal cages were sanitized on a regular schedule. No known contaminants were present in the bedding which could interfere and affect the results of the study. Diet was a commercial, dry rodent chow provided *ad libitum*. Water source was the public supply given *ad libitum*. Mice were killed by CO_2_ exposure at the end of the each study.

For the pharmacokinetic study, 8 healthy female Swiss mice (22–25 g; Charles River Laboratories, Wilmington, MA) were each injected intravenously with 0.125 mL of ^64^Cu-labeled PEG-HAuNS (activity: 20 μCi; 6.25 mg/kg of 50 OD PEG-HAuNS). At predetermined intervals (0 to ~24 h), blood samples (10 μL) were taken from the tail vein, and the radioactivity of each sample was measured with a gamma counter (Packard, Downers Grove, IL). The percentage of the injected dose per gram of blood (%ID/g) was calculated. The blood pharmacokinetic parameters for the radiotracer were analyzed using a noncompartmental model with WinNonlin 5.0.1 software (Pharsight, Palo Alto, CA).

For the biodistribution study, human ovarian cancer tumors were generated by subcutaneous injection of Hey cells (5.0 × 10^6^ cells/mouse, n = 6) in female nu/nu nude mice (Charles River Laboratories). When the average tumor size reached 6–8 mm in average diameter, the mice were injected subcutaneously with 0.125 mL ^64^Cu-labeled PEG-HAuNS (activity: 20 μCi; 6.25 mg/kg of 50 OD PEG-HAuNS). The mice were killed at 24 h after injection. Various tissues, including tumors, were collected and weighed. The radioactivity of each sample was measured with a Cobra gamma counter (Packard Instruments, Downers Grove, IL). Uptake of nanoparticles in various tissues was calculated as %ID/g.

### Antitumor activity

The antitumor activity of PEG-HAuNS at different doses was investigated in female nude mice (Charles River Laboratories) bearing human ovarian tumors. The tumors were generated by subcutaneous injection of Hey cells (5 × 10^6^ cells/mouse). The diameter of tumors was measured by a vernier caliper, and the tumor volume was calculated according to the following equation: volume = (tumor length) × (tumor width)^2^/2. When the tumor volume reached ~300 mm^3^, the mice were divided into 4 groups consisting of 7 or 8 mice/group. Mice in groups 1 to 3 were injected intravenously with a single dose of PEG-HAuNS (5 mL/kg of 25, 50, or 100 OD nanoparticles, corresponding to 3.13 mg/kg, 6.25 mg/kg, or 13.5 mg/kg, respectively). The mice in group 4, as controls, were injected intravenously with a single dose of saline (5 mL/kg). Tumors in all of the groups were irradiated with an NIR laser (2.5 W/cm^2^ for 3 min) at 24 h after drug injection. The tumors were measured as described before. At the end of the experiment (tumors reached >1,500 mm^3^ or 10 days after initial injection, whichever came first), the mice were killed and the tumors removed and weighed.

Human ovarian A2780 tumors were generated by subcutaneous injection of the cells (8.0 × 10^6^ cells/mouse). When tumor volume reached ~500 mm^3^, mice were divided into two groups. Mice in group 1 (n = 6) were injected intravenously with saline (5.0 mL/kg) and mice in group 2 (n = 7) were injected with PEG-HAuNS (5.0 mL/kg of 50 OD; 6.25 mg/kg). Mice in both groups were irradiated with NIR laser (Diomed 15 plus, UK) above the skin surface at 24 h after injection at a power density of 2.5 W/cm^2^ for 3 min). The tumor dimensions were measured with a caliper, and the tumor volume was calculated according to the equation: Volume = (Tumor Length) × (Tumor Width)^2^/2. At the end of the experiment (tumors reached > 2000 mm^3^ or 22 days after initial injection, whichever came first), mice were killed by CO_2_ overexposure and tumors were removed and weighed.

### Acute and chronic toxicity

A total of 24 male and 24 female 8 weeks CD1 mice (Charles River Laboratories) were assigned to 4 groups (6 mice/sex/group). Two groups were injected intravenously with PEG-HAuNS (treated) at a dose of 12.5 mg/kg (accumulated dose: 125 mg/kg) and 2 were injected with saline (controls), for a total of 10 injections, administered daily for 5 injections per week over a 2-week period. At 14 and 90 days from the first injection, 12 mice (6 male, 6 female) were killed. The following tissues were collected at necropsy for histological examination: liver, gall bladder, kidneys, lungs, spleen, skeletal muscle, heart, aorta, adrenal glands, brain, eyes, lacrimal glands, pituitary gland, sciatic nerve, spinal cord, pancreas, stomach, duodenum, jejunum, ileum, cecum, colon, rectum, mesenteric lymph node, salivary glands, mandibular lymph node, thymus, larynx/pharynx with tongue, thyroid gland, parathyroid glands, trachea, esophagus, skin, mammary glands (females only), urinary bladder, female reproductive organs (ovaries, uterus, cervix), male reproductive organs (testes, epididymis, prostate, seminal vessicles), femur with knee joint, sternum, sternal bone marrow, and injection site (tail vein). At the time of necropsy, cardiac blood was collected for hematologic and clinical chemistry analysis, and any gross observations were noted. Selected organs were weighed at the time of necropsy (liver, spleen, kidneys), and relative organ weights were calculated on the basis of the terminal body weights. All tissues were fixed in 10% neutral buffered formalin, processed into paraffin-embedded blocks, and cut into 5-micron sections for histologic examination by a board-certified veterinary pathologist.

To investigate the elimination of PEG-HAuNS in mice, the Au content of liver, spleen, kidney, and muscle samples of 6 CD1 mice (3 male, 3 female) killed at day 14 and day 90 was measured using neutron activation analysis at the University of Missouri Research Reactor Facility (Columbia, MO). Tissue samples were prepared by weighing the as-received samples into precleaned, high-density polyethylene irradiation vials and lyophilizing the tissue to constant dry weight. The samples were then loaded in polyethylene transfer “rabbits” in sets of nine and were irradiated for 90 s in a thermal flux density of approximately 5 × 10^13^ n cm^−2^ s^−1^. The samples were then allowed to decay for 24 to 48 h and counted in real time on a high-purity germanium detector for 3600 s at a sample-to-detector distance of approximately 5 cm. The mass of gold in a sample was quantified by measuring the 411.8 KeV gamma ray from the β^−^ decay of ^198^Au (t_1/2_ = 2.7 days). Six geometrically equivalent comparator standards, prepared by pipetting approximately 0.1 (n = 3) and 0.01 (n = 3) mg of gold from a (10.0 ± 0.5) μg/mL certified standard solution (High-Purity Standards) on paper pulp in the polyethylene irradiation vials, were used with each sample set.

### Statistics

Mean differences in tumor size at the 10^th^ day of treatment between the 4 treatment groups of mice with Hey tumors were analyzed by analysis of variance, with *p* < 0.05 considered to be statistically significant. Difference on tissue concentration between day-14 and day-90 after the first injection was analyzed using student’s *t*-test (paired, two-tail test).

## Competing interest

The authors have no potential competing interest to disclose.

## Authors’ contributions

JY carried out all studies and drafted the manuscript. JZ participated in data analysis and manuscript preparation. MZ participated in biodistribution study. YL participated in the synthesis of PEG-HAuNS. JDR carried out quantitative analysis of tissue concentration of Au. DL carried out pharmacokinetic analysis. CVP designed in vivo toxicity study, carried out tissue histopathological analysis, and drafted the manuscript. CL designed the work and drafted the manuscript. All authors read and approved the final manuscript.

## Supplementary Material

Additional file 1: Table S1 Group means for terminal body weights (TBW) for both the 14-day and 90-day sacrifices and the calculated percent of control for each sacrifice date. **Table S2.** Group means of organ weight relative to body weight and percent of control for both sacrifice dates and sexes. **Table S3.** Group means for both sacrifice dates and both sexes for hematology. **Table S4.** Summary of incidences and average group grades for all microscopic observations. **Figure S1.** Cell viability as a function of PEG-HAuNS concentration. LLC-PK1 (A) and HepG2 (B) cells were treated with PEG-HAuNS for 4, 24, and 48 hr. The viability of cells was determined using the MTT assay. **Figure S2.** Antitumor activity of PEG-HAuNS against A2780 tumor in nude mice. A: A2780 tumor growth curves in mice treated with saline (n = 6) and with PEG-HAuNS (n = 7). All tumors in both groups received NIR laser illumination from the tumor’s surface (2.5 W/cm^2^, for 3 min) at 24 h after intravenous injection of PEG-HAuNS (single, 6.25 mg/kg). B: Average tumor weight in saline- and PEG-HAuNS-treated groups on day 21 after NIR laser illumination. **Figure S3.** Spleen with pigment. The 14-day pigment deposition is much greater than what is observed at 90 days. At 14 days, the pigment deposits were smaller and distributed diffusely throughout the spleen. At 90 days, the deposits were larger and fewer, and focal and random throughout the spleen. Magnification, ×400. Click here for file
