# Supplementary material for: Pharmacokinetics, clearance, and biosafety of polyethylene glycol-coated hollow gold nanospheres
Source: Part Fibre Toxicol. 2014 May 30;11:26. doi: 10.1186/1743-8977-11-26 (PMC4082425; doi:10.1186/1743-8977-11-26)
Supplement: Additional file 1: Table S1 — Group means for terminal body weights (TBW) for both the 14-day and 90-day sacrifices and the calculated percent of control for each sacrifice date. Table S2. Group means of organ weight relative to body weight and percent of control for both sacrifice dates and sexes. Table S3. Group means for both sacrifice dates and both sexes for hematology. Table S4. Summary of incidences and average group grades for all microscopic observations. Figure S1. Cell viability as a function of PEG-HAuNS concentration. LLC-PK1 (A) and HepG2 (B) cells were treated with PEG-HAuNS for 4, 24, and 48 hr. The viability of cells was determined using the MTT assay. Figure S2. Antitumor activity of PEG-HAuNS against A2780 tumor in nude mice. A: A2780 tumor growth curves in mice treated with saline (n = 6) and with PEG-HAuNS (n = 7). All tumors in both groups received NIR laser illumination from the tumor’s surface (2.5 W/cm2, for 3 min) at 24 h after intravenous injection of PEG-HAuNS (single, 6.25 mg/kg). B: Average tumor weight in saline- and PEG-HAuNS-treated groups on day 21 after NIR laser illumination. Figure S3. Spleen with pigment. The 14-day pigment deposition is much greater than what is observed at 90 days. At 14 days, the pigment deposits were smaller and distributed diffusely throughout the spleen. At 90 days, the deposits were larger and fewer, and focal and random throughout the spleen. Magnification, ×400. [file 1743-8977-11-26-S1.pdf]

Supplemental Information

Pharmacokinetics, Clearance, and Biosafety of Polyethylene Glycol-Coated Hollow Gold Nanospheres

Jian You[1], Jialin Zhou[1], Ming Zhou[2], J. David Robertson[3], Dong Liang[4], Carolyn Van Pelt[5*], Chun Li[2*]

[1]College of Pharmaceutical Sciences, Zhejiang University, Yuhangtang Road 388, Hangzhou 310058, People's Republic of China.

[2]Department of Cancer Systems Imaging and [5]Department of Veterinary Medicine and Surgery, The University of Texas MD Anderson Cancer Center, Houston, Texas 77030, United States

[3]Department of Chemistry, University of Missouri-Columbia, Columbia, Missouri 65211, United States

[4]Department of Pharmaceutical Sciences, College of Pharmacy and Health Sciences, Texas Southern University, 3100 Cleburne Street, Houston, TX 77004, United States

[5]Department of Veterinary Medicine and Surgery, The University of Texas MD Anderson Cancer Center, Houston, 77030, TX, United States

Table S1. Group means for terminal body weights (TBW) for both the 14-day and 90-day sacrifices and the calculated percent of control for each sacrifice date

Group	Terminal body weights (g)			
	14 days (acute toxicity)		90 days (chronic toxicity)	
	Male	Female	Male	Female
Saline	36.18	28.61	49.66	40.34
PEG-HAuNS	34.01	26.70	47.08	42.24
% of control *	94.0	93.3	94.8	104.7

* % of control = $(TBW_{PEG\text{-}HAuNS} / TBW_{Saline}) \times 100$

Table S2. Group means of organ weight relative to body weight and percent of control for both sacrifice dates and sexes.

Group	Organ	Relative organ wts *(%)			
		14 days (acute toxicity)		90 days (chronic toxicity)	
		Male	Female	Male	Female
Saline	Liver	5.3	4.51	5.6	4.39
	Kidney	1.68	1.3	1.58	1.27
	Spleen	0.39	0.48	0.42	0.51
PEG-HAuNS	Liver	5.33	5.07	5.83	4.09
	Kidney	1.69	1.32	1.57	1.25
	Spleen	0.46	0.57	0.34	0.48
% of control	Liver	100.6	112.4	104.1	93.2
	Kidney	100.6	101.5	99.4	98.4
	Spleen	117.9	118.8	81.9	94.1

* Relative organ wts = (organ weight/TBW) × 100

Table S3. Group means for both sacrifice dates and both sexes for hematology

Parameter*	Unit	Female				Male				Reference ranges	
		14 days		90 days		14 days		90 days		6-8-week Swiss mice	
		Saline	PEG-HAuNS	Saline	PEG-HAuNS	Saline	PEG-HAuNS	Saline	PEG-HAuNS	Male	Female
HGB	g/dl	15.7	15.8	14.9	14.3	15.7	15.9	16.1	15.2	12.2-15.1	12.1-16.5
HCT	%	51.5	50.9	49.5	47.9	50.9	52.3	53.2	50.5	36.7-44.2	36.2-48.2
WBC	10^3/ul	6.2	11.08 **	7.42	6.79	8.52	8.05	8.03	5.89	1.9-7.2	2.1-7.1
RBC	10^6/ul	10.26	10.15	9.84	9.79	10.39	10.69	10.58	10.2	7.1-9.3	7.4-9.9
MCV	fl	50.2	50.2	50.4	48.9	49.1	48.9	50.3	49.4	45.8-52.5	46.0-51.4
MCH	pg	15.3	15.5	15.1	14.7	15.1	14.9	15.2	14.9	15.5-17.8	15.5-17.5
MCHC	g/dl	30.5	30.9	30.1	30.1	30.8	30.4	30.3	30.1	32.5-35.1	32.5-35.4
PLATELETS	10^3/ul	1335	1299	1281	1063	1602	1320	1318	1469	543.6-1854.1	659-1427
SEGS	%	9.6	9.9	13.2	13.2	14.7	13.1	14.5	18.6	7.7-24.3	7.4-25.9
LYMPHS	%	85.6	83.3	79.1	79.8	79	80.1	75	71.3	62.6-87.8	60.8-85.0
MONOS	%	2.2	1.8	2.9	2.7	3.3	3.1	4.4	4.8	0.3-4.1	0.2-4.4
EOS	%	1.1	2.7	2.4	2.5	1.7	1.6	3.8	3.4	0.0-5.6	0.0-13.0
BASOS	%	0.3	0.3	0.4	0.2	0.2	0.2	0.3	0.3	0.0-0.8	0.0-0.7
LUC	%	1.2	2	2.2	1.4	1.2	2.7	2	1.7	0.0-13.8	0.0-7.6
UMC					2		0.5			0	0

SEGS	10^3/ul	0.59	1.09	0.92	0.83	1.26	1.01	1.11	1.05	0.3-1.1	0.2 - 1.3
LYMPHS	10^3/ul	5.31	**9.23** **	5.95	5.51	6.72	6.48	6.04	4.22	1.1-5.8	1.5-5.3
MONOS	10^3/ul	0.14	0.21	0.21	0.18	0.28	0.25	0.35	0.29	0.0-0.2	0.01-0.20
EOS	10^3/ul	0.07	**0.31** **	0.16	0.15	0.15	0.17	0.34	0.22	0.01-0.21	0.0-0.68
BASOS	10^3/ul	0.02	0.03	0.03	0.01	0.02	0.02	0.03	0.01	0.0-0.03	0.0-0.04
LUC	10^3/ul	0.08	0.21	0.15	0.09	0.09	0.21	0.16	0.09	0.0-0.6	0.0-0.40
UMC	10^3/ul				0.17		0.06			0	0

* HGB- hemoglobin, HCT- hematocrit, WBC- white blood count, RBC - red blood count, MCV - erythrocyte mean corpuscular volume, MCH - mean corpuscular hemoglobin, MCHC - mean corpuscular hemoglobin concentration, SEGS - segmented neutrophils, LYMPHS - lymphocytes, MONOS - monocytes, EOS - eosinophil, BASOS - basophils, LUC - large unstained cell, UMC - unclassified mononuclear cell.

**Alterations from the respective control group.

Table S4. Summary of incidences and average group grades for all microscopic observations

		Control				PEG-HAuNS				Control				PEG-HAuNS			
	Time (day)	14								90							
		F		M		F		M		F		M		F		M	
		I	G	I	G	I	G	I	G	I	G	I	G	I	G	I	G
**Liver**																	
Pigment	Kupffer Cells	0/6	0	0/6	0	6/6	4	6/6	3.7	0/6	0	0/6	0	6/6	3.3	6/6	3.6
	Hepatocytes	0/6	0	0/6	0	0/6	0	2/6	1.5	0/6	0	0/6	0	4/6	1.3	0/6	0
Inflammation	Purulent	0/6	0	1/6	1	0/6	0	5/6	1.2	0/6	0	1/6	1	0/6	0	3/6	1
	Mononuclear	2/6	1	0/6	0	6/6	1.7	0/6	0	0/6	0	2/6	1	5/6	1.6	6/6	1.9
Necrosis, focal	Necrosis	0/6	0	0/6	0	0/6	0	3/6	1	0/6	0	1/6	1	2/6	1	0/6	0
Increased Mitosis of Hepatocytes	Mitosis	0/6	0	0/6	0	1/6	1	1/6	1	0/6	0	0/6	0	0/6	0	1/6	1
**Lung**																	
Inflammation/hitiocytic/ granuloma	Granuloma	0/6	0	0/6	0	1/6	1	1/6	1	0/6	0	0/6	0	0/6	0	2/6	1
	Histiocytosis, interstitial	0/6	0	0/6	0	2/6	1	1/6	1	0/6	0	0/6	0	0/6	0	0/6	0
	Histiocytosis, peribronchiolar	0/6	0	0/6	0	0/6	0	0/6	0	0/6	0	0/6	0	1/6	1	0/6	0
	Histiocytosis, perivascular	0/6	0	0/6	0	1/6	1	0/6	0	0/6	0	0/6	0	0/6	0	0/6	0
	Histiocytosis, lobular	0/6	0	0/6	0	1/6	3	0/6	0	0/6	0	0/6	0	0/6	0	0/6	0
	Summary, histiocytosis, granuloma	0/6	0	0/6	0	5/6	1.7	2/6	1	0/6	0	0/6	0	1/6	1	2/6	1
Pigment	Perivascular	0/6	0	0/6	0	1/6	1	0/6	0	0/6	0	0/6	0	0/6	0	1/6	1
	TB lymph node	0/6	0	0/6	0	0/6	0	0/6	0	0/6	0	0/6	0	0/6	0	1/6	1
**Spleen**																	
Pigment, macrophages		0/6	0	0/6	0	2/6	1	2/6	1.5	0/6	0	0/6	0	6/6	1.5	6/6	2
Follicular Lymphoid Hyperplasia		5/6	1.8	4/6	2	6/6	2.3	6/6	2.7	4/6	1	6/6	2.2	6/6	1.2	5/6	1.6

Extramedullary Hematopoiesis		6/6	2.2	2/6	2	4/6	1.3	1/6	1	5/6	1.6	5/6	1.6	6/6	2.2	6/6	2.7
**Heart**																	
Pigment	Subendocardial	0/6	0	0/6	0	1/6	1	1/6	1	0/6	0	0/6	0	0/6	0	0/6	0
	Perivascular	0/6	0	0/6	0	1/6	1	0/6	0	0/6	0	0/6	0	2/6	1	1/6	1
	Cardiomyocytes	0/6	0	0/6	0	0/6	0	0/6	0	0/6	0	0/6	0	0/6	0	1/6	1
	Summary, Pigment	0/6	0	0/6	0	2/6	1	1/6	1	0/6	0	0/6	0	2/6	1	2/6	1
Cardiomyopathy/ Myocardiocyte	Cardiomyopathy	0/6	0	0/6	0	0/6	0	0/6	0	0/6	0	2/6	1	0/6	0	0/6	0
degeneration/ inflammation/fibrosis	Myocardiocyte degeneration	0/6	0	0/6	0	0/6	0	0/6	0	0/6	0	0/6	0	1/6	1	0/6	0
	Inflammation, focal, mononuclear	0/6	0	0/6	0	0/6	0	0/6	0	0/6	0	1/6	1	0/6	0	1/6	1
	Fibrosis, Subendocardial	0/6	0	0/6	0	0/6	0	1/6	2	0/6	0	0/6	0	0/6	0	0/6	0
	Summary: Cardiomyopathy	0/6	0	0/6	0	0/6	0	1/6	2	0/6	0	3/6	1	1/6	1	1/6	1
**Adrenal Gland** (Females only)																	
Vacuolation/ pigment, cortex	Vacuolation	0/6	0	-	-	4/6	1.5	-	-	4/5	2.8	-	-	2/6	3	-	-
	Pigment	0/6	0	-	-	1/6	1	-	-	0/5	0	-	-	2/6	1	-	-
**Injection Site** (Tail Vein)																	
Inflammation, perivascular/vascular wall	Perivascular	2/6	1	2/6	1.5	6/6	2	5/5	1.6	0/6	0	1/6	1	0/6	0	0/6	0
	Vascular wall	0/6	0	0/6	0	3/6	1	0/5	0	0/6	0	0/6	0	0/6	0	0/6	0
	Summary, Inflammation	2/6	1	2/6	1.5	6/6	2.5	5/5	1.6	0/6	0	1/6	1	0/6	0	0/6	0
Pigment, perivascular/vascular wall	Perivascular	0/6	0	0/6	0	0/6	0	1/5	2	0/6	0	0/6	0	0/6	0	1/6	1
	Vascular wall	0/6	0	0/6	0	1/6	1	0/5	0	0/6	0	0/6	0	0/6	0	0/6	0
	Pigment Summary	0/6	0	0/6	0	1/6	1	1/5	2	0/6	0	0/6	0	0/6	0	1/6	1

F = Female; M = Male; I = Incidence, expressed as incidence number over examined number. For example, 0/6 means 6 samples were examined and there were 0 incidences. G - Group Grade. Grading key: Grade 1 = modest, rare 5-10% of tissue affected; Grade 2 = mild, slight, infrequent 10-20% of tissue affected; Grade 3 = moderate, frequent 20-50% of tissue affected; Grade 4 = severe, extensive >50% of tissue affected.

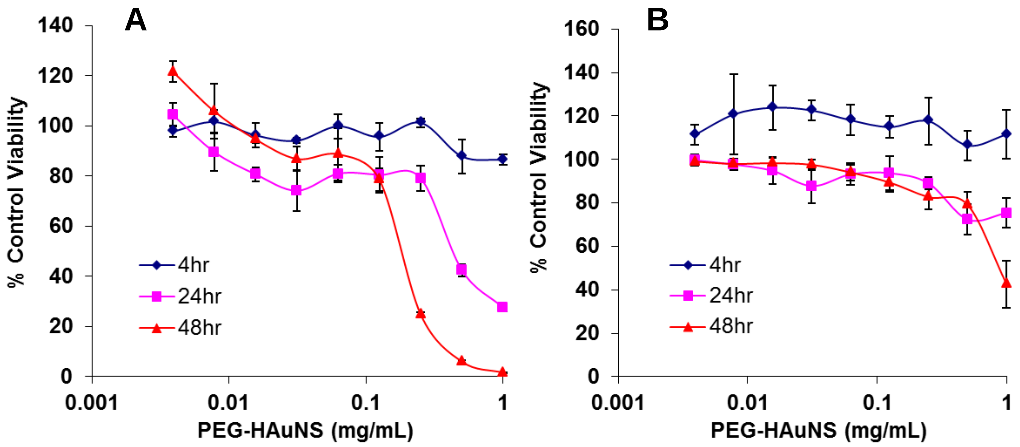

Fig. S1. Cell viability as a function of PEG-HAuNS concentration. LLC-PK1 (A) and HepG2 (B) cells were treated with PEG-HAuNS for 4, 24, and 48 hr. The viability of cells was determined using the MTT assay.

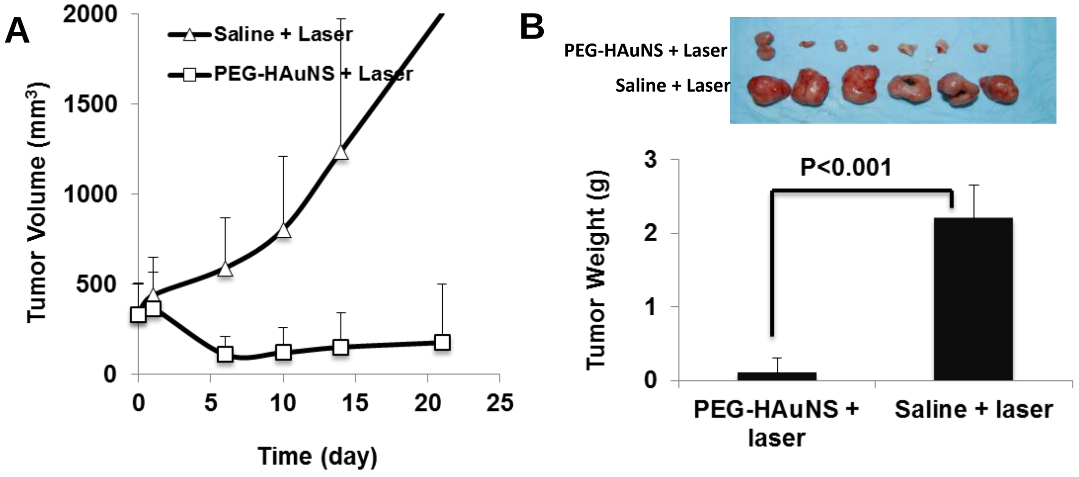

Fig. S2. Antitumor activity of PEG-HAuNS against A2780 tumor in nude mice. **A**: A2780 tumor growth curves in mice treated with saline (n = 6) and with PEG-HAuNS (n = 7). All tumors in both groups received NIR laser illumination from the tumor's surface (2.5 W/cm^2, for 3 min) at 24 h after intravenous injection of PEG-HAuNS (single, 6.25 mg/kg). **B**: Average tumor weight in saline- and PEG-HAuNS-treated groups on day 21 after NIR laser illumination.

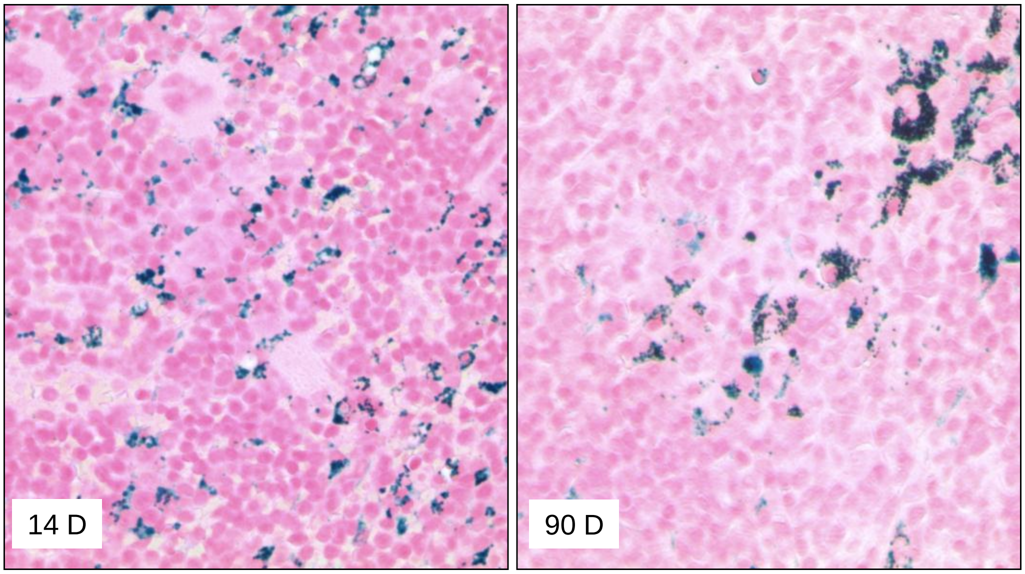

Fig. S3. Spleen with pigment. The 14-day pigment deposition is much greater than what is observed at 90 days. At 14 days, the pigment deposits were smaller and distributed diffusely throughout the spleen. At 90 days, the deposits were larger and fewer, and focal and random throughout the spleen. Magnification, x400.
